# Dendritic cells and HIV transmission: roles and subsets of antigen-presenting cells in the human anogenital tract

**DOI:** 10.1371/journal.ppat.1013490

**Published:** 2025-09-10

**Authors:** Daniel J. Buffa, Thomas R. O’Neil, Erica E. Vine, Lara Sarkawt, Freja A. Warner van Dijk, Oscar A. Dong, Najla Nasr, Anthony L. Cunningham, Kirstie M. Bertram, Andrew N. Harman

**Affiliations:** 1 Centre for Virus Research, The Westmead Institute for Medical Research, Westmead, Australia; 2 School of Medical Sciences, Faculty of Medicine and Health, The University of Sydney, Sydney, Australia; University of North Carolina at Chapel Hill, UNITED STATES OF AMERICA

## Abstract

Dendritic cells (DCs) are potent antigen-presenting cells and play a key role in facilitating the sexual transmission of HIV, functioning as a delivery system responsible for trafficking the virus from exposed barrier sites to their key target cells, CD4 T cells. Although the role of DCs in HIV transmission is well established, the recent advent of high-parameter, single-cell detection technologies, coupled with improved cell isolation techniques, has led to the rapid reclassification of the DC landscape, particularly within human barrier tissues. The identification of new subsets introduces the challenge of incorporating previously understood transmission principles with new, cell-specific, functional nuances to identify the key DCs responsible for facilitating HIV infection. This review explores the history of research linking DCs with HIV transmission as well as our understanding of how HIV manipulates DC biology to achieve this purpose. Furthermore, it provides an up-to-date understanding of the antigen-presenting cell landscape within human anogenital tissues and how each subset contributes to sexual transmission. Uncovering the cells and biological processes responsible for the sexual transmission of HIV is a fundamental step in the pursuit of an HIV vaccine and better prophylaxis to block infection.

## Introduction

HIV’s primary target is CD4 T cells. In activated CD4 T cells, HIV hijacks the cell’s replicatory machinery to drive viral replication and *de novo* virus production, eventually resulting in cell lysis and the release of virions—perpetuating the virus life cycle [1–3]. In resting memory CD4 T cell subsets, HIV can establish latent infection, which cannot be eradicated by antiretroviral treatments [4,5]. Despite targeting CD4 T cells, HIV is unlikely to naturally encounter these cells during transmission as they are infrequently present at the epithelial surface of human tissues, instead relying on antigen-presenting cells (APC), especially dendritic cells (DC), and Langerhans cells (LC) to deliver the virus to them. LCs and DCs were identified as targets for HIV infection concurrently as the pandemic erupted, and our understanding of how these cells facilitate transmission has steadily evolved since. Here, we review: our current understanding of the various subsets of DCs and LCs; the mechanisms by which they transmit the virus to CD4 T cells; how HIV manipulates their biology, and which specific subsets play a dominant role in transmitting HIV to T cells. The main emphasis of discussion focuses on the *bona fide* DC/LC subsets that are present in steady-state (noninflamed) human anogenital tissues, which represent the first cells to encounter the virus during sexual transmission [6,7].

## Historic identification of Langerhans cells and dendritic cells

LCs were first observed by Paul Langerhans in 1868 [8], who classified them as an epidermal nerve cell due to their dendritic-like morphology. The field then lay dormant for over a century until 1973, when Ralph Steinman at Rockefeller University discovered a murine splenic cell with a branching, dendritic morphology—the Dendritic Cell [9]. At the time, macrophages were believed to be the key cells that initiated adaptive immunity [10–12]. However, Steinman and colleagues showed that DCs were the key ‘accessory cell’ responsible for linking innate and adaptive immunity [9], a discovery that earned him a Nobel Prize in 2011, three days after his passing.

Steinman demonstrated that DCs were the most potent immune cells at initiating T cell responses through their ability to take up, process and then present antigens to naïve T cells via major histocompatibility complex (MHC) molecules [13,14]. Initial experiments uncovered the foundational, functional properties of DCs and cemented them as the key APC to T cells. Steinman’s discoveries led to a rapid expansion in DC research. Epidermal LCs resurfaced as a key skin APC in the late 1970s as they were also shown to express MHC molecules and stimulate robust T cell responses [15,16], leading to their classification as a DC subset. Moreover, the residence of LCs in the epidermis made them the first DCs to be observed in human barrier tissues.

In the 1990s, DCs were split into two classes. Firstly, the Steinman group demonstrated that certain DCs are derived from myeloid precursors and named them conventional DCs (cDC) [17]. Secondly, lymphatic-derived DCs were identified in blood and lymphoid organs and subsequently named plasmacytoid DCs (pDC) [18–20]. In addition to postulated APC capabilities [18], pDCs play a unique role in antiviral responses by producing large amounts of type I interferons (IFN) [19–22]. In 1994, Federica Sallusto and Antonio Lanzavecchia discovered that circulating blood monocytes could differentiate into DC-like cells upon *in vitro* culture with cytokines, particularly GM-CSF and IL-4 [23,24]. The relative ease of generating these cultured monocyte-derived DCs (*in vitro* MDDC) meant that their use in functional experiments, including HIV assays, dominated the literature for many decades. During the 2000s, a tissue-resident, *in vivo* monocyte-derived DC (*in vivo* MDDC) was identified when human tissue CD14+ cells, previously classified as macrophages, were found to comprise an autofluorescent macrophage population and a nonautofluorescent DC population [25]. It is now clear that *in vitro* MDDCs differ significantly from *in vivo* MDDCs that reside within tissue [26,27]. By the end of the 2000s, DCs were therefore subdivided into five classes based on ontogeny, function and/or tissue residency: LCs, cDCs, pDCs, *in vitro* derived MDDCs, and *in vivo* MDDCs.

## Evolving classifications and unification of contemporary APC subsets

Many new APC subsets have been described over the past 25 years, often based on limited surface marker expression and/or tissue location. However, the advent of high-parameter, single-cell technologies, especially RNA sequencing (RNA-seq) has not only led to the identification of many new APC subsets but has also led to consensus on what the key human APC subsets are. Firstly, there are currently considered to be three subsets of bone marrow-derived cDCs—DC1–3. DC1s are functionally specialised for cross-presentation of antigens to CD8 T cells, DC2s present antigens mainly to CD4 T cells and early research suggests that blood DC3s may be capable of both CD4 and CD8 activation [28–35]. Secondly, in 2014, what was previously classified as an *in vivo* CD14^+^ MDDC was shown to, in fact, represent a monocyte-derived macrophage (MDM) [36]. However, it is now known that tissue nonautofluorescent CD14^+^ immune cells represent a heterogeneous population of *in vivo* MDMs and MDDCs that can be differentiated by dendritic cell-like markers, such as CD1c and CD11c [26,37,38]. Finally, LC classification has undergone several revisions prior to its contemporary definition as a *bona fide* APC population. LCs ontologically align with macrophages, as they are seeded during embryogenesis and are mostly maintained through self-replication within barrier tissues [39–41]. Whilst still possessing some macrophage functional traits, LCs' primary APC capabilities renders their functionality closer to DCs [42,43]. Given this duality, LCs are referred to as both DCs and macrophages in the literature, but are best classified as their own class of APC. In 2021, Liu and colleagues defined four LC subsets (LC1–4) within human foreskin epidermis with LC3 and LC4 being combinations of LC1 and LC2 in different functional states [44]. However, LC2 express DC delineating markers such as CD1c and there are conflicting opinions as to whether LC2 are a *bona fide* LC population, or if they are the newly defined epidermal/epithelial DCs [45–47].

## Anogenital barrier tissues

Although HIV can be transmitted via an exchange of blood, such as from mother to child during birth or intravenous drug use, sexual transmission is by far the predominant transmission mechanism. The tissue sites most associated with sexual transmission of HIV are therefore those that comprise anorectal and genital (anogenital) tracts, which consist of three tissue types. Firstly, **skin** covers the glans penis, labia, anal verge, and outer foreskin. Skin contains a highly keratinised stratified squamous epithelium (SSE), consisting superficially of epidermis, which forms a tough physical barrier to pathogen entry. Immediately deep to the epidermis are two layers of connective tissue: the superficial papillary layer and the deeper reticular layer of the dermis. Moving proximally along the anogenital tracts, skin transitions into the **type II mucosa** of the vagina, ectocervix, inner foreskin, fossa navicularis, and anal canal, which consists of a non- or thinly keratinised SSE, and underlying lamina propria. The minimal or lack of epidermal keratinisation makes it a weaker barrier to pathogens [48–52]. Moving further internally, the final barrier tissue associated with the sexual transmission of HIV is **type I mucosa**, covering the endocervix, penile urethra, and colorectum. This consists of a single columnar epithelium overlying two layers of connective tissue, the superficial lamina propria and the deeper submucosa. Within these type I mucosal tissue layers are lymphocyte-rich, tertiary lymphoid structures known as lymphoid follicles/aggregates. As discussed below, the different tissue compartments of the anogenital tracts contain distinct DC subsets, with the tissue microenvironment known to drive phenotypical changes between them.

## The role of antigen-presenting cells in mediating HIV transmission

**Fig 1:**
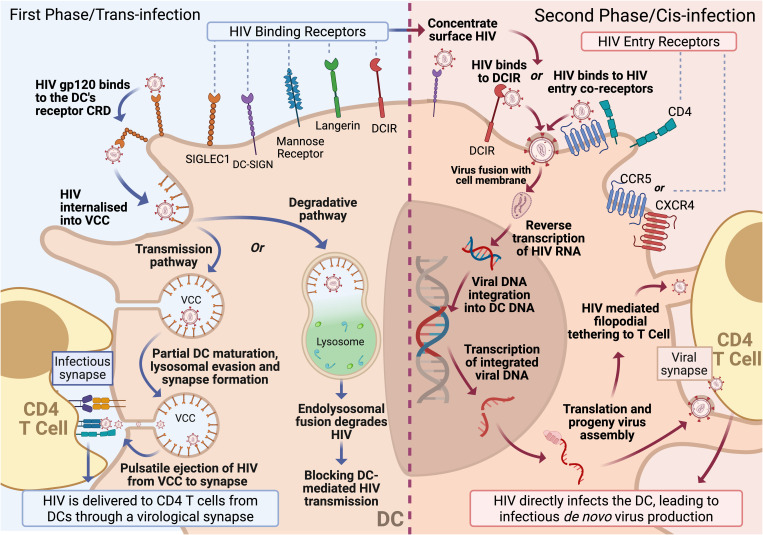
Biphasic HIV trafficking through dendritic cells to CD4 T cells. DC-mediated HIV transmission occurs through two mechanisms**. First phase or trans-infection** is initiated by binding of virus to specific HIV lectin ‘binding receptors’, which rapidly internalises/endocytoses the virus into a nondegradative, virus-containing cave (VCC). Following VCC formation, the virus can undertake two pathways. Firstly, the transmission pathway leads to partial DC maturation, immune and viral degradation evasion, and transport/migration to CD4 T cells. First-phase transmission concludes with the formation of an infectious synapse between the DC and CD4 T cell, where the virus is released from the VCC to the CD4 T cell. The degradation pathways result in the destruction of the virus through the fusion of VCCs with lysosomes. **Second phase or *cis*-infection** begins with viral fusion of HIV with the DC membrane, mediated by the binding of virions to the HIV entry receptor, CD4, and its co-receptor CCR5. HIV binding lectin receptors such as Siglec-1, DC-SIGN, and DCIR can also concentrate the virus on the DC surface, leading to CD4/CCR5-mediated HIV infection. Once within the cell, the viral RNA is reverse transcribed to cDNA, which is then transported to the nucleus and integrated into the host cell genome. The virus then hijacks the cells replicatory machinery to produce and release progeny virions. Accompanying *de novo* virus production, productive infection also enhances T cell–dendritic cell interactions by driving the extension of filopodia that tether to T cells. This facilitates the direct transfer of budding virus to the tethered T cell via a viral synapse, culminating in CD4+ T cell infection and the continued perpetuation of the viral lifecycle. Figures created with Biorender.com.

As both LCs and DCs are potent APCs present within the most superficial layers of distal human barrier tissues, they are the ideal vehicles to deliver the virus from the sites of viral exposure to CD4 T cells, which are the primary HIV target cell responsible for HIV replication. The first indication that DCs may facilitate viral delivery followed observations that co-cultures of T cells, *in vitro*-derived MDDCs and HIV drove greater rates of CD4 T cell infection compared to similar co-cultures without MDDCs [53]. In 2000, Geijtenbeek and colleagues described a mechanism by which HIV binds to dendritic cell-specific intercellular adhesion molecule-3-grabbing nonintegrin (DC-SIGN) on *in vitro* MDDCs—subsequently facilitating transfer to T cells [54]. Thus, suggesting a pathway in which HIV harnesses DCs antigen presentation capabilities to reach its primary target. Shortly thereafter, it became apparent that other C-type lectin receptors, such as langerin and mannose receptor, were efficient at HIV binding in epidermal DCs/LCs [55,56]. Additionally, DCs were found to transmit HIV to CD4 T cells via two independent mechanisms known as first-phase transfer (*trans-*infection) and second-phase transfer (*cis-*infection) [57,58] (Fig 1).

### First-phase transfer/*trans*-infection

First-phase transfer is facilitated by membrane-bound lectin receptors on the DC/LC surface. These receptors include C-type Lectin Receptors (CLR) which bind oligosaccharides on the envelope protein (gp120) with high affinity [59] and Sialic Acid Binding Immunoglobulin-Type Lectin-1 (Siglec-1) which binds gangliosides within the virus envelope in addition to mannose residues on gp120 [60,61]. After binding to HIV-associated lectin receptors, the virus is internalised/endocytosed into protective virus-containing caves (VCCs) [60,62,63]. These VCCs are neutral pH, continuous with the plasma membrane and when the DC interacts with CD4 T cells, an infectious synapse (morphologically similar to an immunological synapse but functionally distinct [64–66]) forms between the two, whereby the virus is pulsed into the protected intercellular gap allowing CD4 T cells to become infected [57,62]. First-phase transfer begins to occur within two hours of exposure of DCs/LCs to HIV and rapidly declines over 24 h, by which time no transfer occurs, presumably because the virions contained within VCCs are degraded through endolysosomal processing, by a mechanism yet to be determined [58,67–69].

Five HIV-binding lectin receptors have been identified to date: CD209/DC-SIGN, CD169/Siglec-1, CD206/Mannose Receptor (MR), CD207/langerin, and CD367/Dendritic Cell ImmunoReceptor (DCIR).

DC-SIGN/CD209 was the first receptor shown to bind HIV and has been extensively studied [54]. DC-SIGN directs the virus into VCCs and also binds to ICAM-3 on T cells, stabilising the physical connection between MDDCs and T cells [54,70]. Despite early conjecture that DC-SIGN was specifically expressed by DCs, recent studies show that skin/mucosal DCs in human steady-state tissue do not express this receptor (including *in vivo* MDDCs) [26,49]. Interestingly, DC-SIGN is only expressed by macrophages in steady-state tissue but can be expressed by *in vivo* MDDCs in inflamed tissues [26,56,71]. Although macrophages can stimulate already primed T cells, they are weak at priming *de novo* T cell responses [72]. However, Rhodes and colleagues showed that they can facilitate HIV transfer to CD4 T cells in human anogenital tissues, albeit less efficiently than DCs [26]. The specific role of macrophages in HIV transmission requires further attention, particularly given the continual discovery of new discrete subsets, some of which express DC-associated receptors such as CD11c on Mf2s [73–77].

SIGLEC-1/CD169 expression is induced by IFN-α signalling [78,79] and has been more recently identified as an HIV uptake receptor. Like DC-SIGN, it efficiently traffics the virus into VCCs, plays a direct role mediating transfer of HIV CD4 T-cells [80,81], and is most highly expressed by macrophages. *In vivo* MDDCs also express SIGLEC-1 but at much lower levels than macrophages [26]. Additionally, recent literature suggests that an inflammatory subset of DCs, the ASDC, also expresses SIGLEC-1 [21,82]. Interestingly, Perez-Zsolt et al. have also recently demonstrated that cervical *in vivo* derived MDDCs expressed Siglec-1 and demonstrated that blocking HIV from binding SIGLEC-1 resulted in decreased infection and transfer to CD4 T cells, however, this is likely a mixed population of *in vivo* MDDCs and MDMs [38].

Langerin/CD207 is expressed very highly by LCs and was long thought to be an LC-distinguishing molecule, thus, its role as an HIV uptake CLR has been heavily investigated [83]. LCs also contain Birbeck granules which are composed of oligomeric langerin. As discussed below, there is significant controversy regarding the role of LCs in HIV infection [84]. However, it is clear that on primary tissue isolated LCs, langerin binds HIV and mediates endocytic uptake and transfer to T cells [83,85]. Importantly, langerin has since been shown to be expressed by some tissue-resident DCs, with notably increased expression observed on DCs residing within anogenital mucosa. However, these cells express langerin at approximately 10-fold lower levels than LCs and it is not clear if these cells bind HIV using this CLR [26,49,71].

DCIR/CD367 is a CLR expressed on numerous DC subsets, including tissue-resident DCs at sexual transmission exposure sites, however, it is less potent at facilitating first-phase transfer to T cells compared to DC-SIGN [26,86]. Nevertheless, blocking the DCIR carbohydrate recognition domain on *in vitro* MDDCs reduces HIV transfer to T cells [86–88]. Interestingly, DCIR has also been strongly implicated in second-phase transfer, as discussed below.

Mannose Receptor/CD206 mediates HIV uptake in both a calcium dependent and independent manner [89]. Early studies postulated that MR could play a significant role in HIV transmission, as it was shown that MR-expressing, *in vitro* MDMs were highly capable of binding and transferring HIV to T cells [90]. However, subsequent research suggests that DC-specific MR binding to whole HIV virions is weak and that following attachment to MR, the virus is rapidly trafficked to lysosomes, where it is degraded [55,89]. More recent literature regarding MR’s role in HIV transmission has focussed on its involvement in macrophage-mediated HIV infection. Two recent studies have found that MR functions as an HIV restriction factor when expressed by macrophages, with HIV-1 counteracting this restriction in infected cells by transcriptionally silencing *MR* expression [91,92].

### Second-phase transfer/*cis*-infection

The second mechanism by which DCs/LCs mediate transfer of HIV to T cells is called **second-phase transfer (or *cis*-infection)** and begins 48–72 h post-HIV exposure. This is dependent on productive APC infection. This process involves the fusion of the virus envelope with the APC plasma membrane, mediated by gp120 binding to the CD4 entry receptor and CCR5 or CXCR4 co-receptor [93,94]. The co-receptor involved in internalisation is dependent on HIV tropism, with R5 strains co-binding CCR5, X4 strains binding to CXCR4, and dual-tropic viruses able to utilise both [95,96] (Fig 1). R5 strains represent the dominant tropism associated with DC infection, as well as with transmission strains [97–99].

Although lectin receptors are predominantly involved in endocytic uptake and associated first-phase transfer, they can also concentrate the virus on the cell surface, leading to infection via CD4 and CCR5/CXCR4. For example, Siglec-1 has been shown to catalyse productive infection, by stabilising interactions between HIV and both CCR5 and CXCR4 [82]. Similarly, DC-SIGN has been shown to bind to HIV surface envelope protein gp120, this physical connection has been shown to enhance HIV entry in a CD4-dependent manner [100,101]. Furthermore, DCIR has been shown to enhance productive infection of immature MDDCs, leading to increased *de novo* virus production [60,87]. Transfer to CD4 T cells is facilitated by HIV stimulated induction of the enzyme Diaphanous 2 and subsequent actin polymerisation. This actin polymerisation drives the extension of filopodia, which bear HIV at their tips, towards CD4 T cells. These filopodia contact and capture CD4 T cells, leading to viral synapse formation between them, releasing the budding HIV virions into the synapse for passage to CD4 T cells, leading to their infection [102]. As second-phase transfer relies on the canonical steps of HIV replication, it can be blocked within the DC by current prophylactics that restrict virus entry, reverse transcription, integration, and virion maturation [49,85,103,104]. DC subsets are also known to express varying amounts of HIV restriction factors, including SAMHD1 and APOBEC3G. Currently, there are two described mechanisms by which SAMHD1 restricts virus propagation. Firstly, SAMHD1 has been shown to hydrolyse dNTPs, critical for HIV reverse transcription and thus blocks viral replication [105]. Secondly, recent literature suggests that SAMHD1 can block viral integration through a non-dNTP-related restriction mechanism. This involves the binding of SAMHD1 molecules to intracellular myxovirus resistance protein B (MxB), to form a tandem trap which binds to and captures the viral capsid. This capsid trap restricts viral interactions with the nucleus, essentially trapping the viral genome within the cytoplasm. However, this second mechanism has only been observed using *in vitro* MDMs and human-derived DC cell lines [106–108]. APOBEC3G also restricts DC infection by two mechanisms. Firstly, by driving hypermutations to the viral genome through cytidine deaminase activity—altering the replicated viral genome and rendering it ineffective [109–111]. Secondly, by deaminase-independent, synthesis inhibition of HIV cDNA/reverse transcripts [111–114]. Therefore, DC subsets that express higher levels of restriction factors are less efficient at mediating second-phase transfer. Notably, DCs express the highest levels of these restriction factors and cannot become infected by HIV or transfer the virus to CD4 T cells.

Following productive infection of the DC, traditional literature suggests that HIV-infected DCs are cleared by CD8 T cells, Natural Killer cells (NK), or through virus-driven cytotoxicity and restriction factors. However, emerging evidence indicates that HIV can modulate DCs to evade these mechanisms, with tissue-resident DCs now recognised as potential long-lived viral reservoirs [115–117]. Firstly, evidence suggests that HIV driven cellular manipulations can help mask infected DCs from both T cells and NK cells [115,118–120]. These processes may help explain recent findings by Banga and colleagues who demonstrated that both migratory and resident lymph node DCs can harbour replication-competent HIV for over 14 years, despite ART suppression, with viral production reactivated upon TLR7/8 stimulation [115]. Collectively, recent evidence suggesting that lymph nodes may serve as a viral reservoir combined with studies describing their distinct immune cell populations, positions lymph nodes as a key tissue site for future investigation in persistent HIV infection [116,121,122]. Ultimately, the broad and complex role of DCs as HIV reservoirs extends beyond the scope of this review, instead, we direct readers to a recent review by Banga and colleagues covering this topic [116].

## Manipulation of antigen-presenting cell biology by HIV

Although DCs and LCs are ontologically distinct, they possess similar functional characteristics as efficient APCs. Upon pathogen uptake, DCs/LCs undergo a generic maturation process which is marked by the differential expression of several surface molecules [123,124]. Firstly, pathogen-binding lectin receptor expression is decreased [26,123]. Secondly, molecules associated with chemotaxis and lymphatic drainage are increased, particularly CCR7, which mediates migration to T cell-rich lymph nodes along CCL19 and CCL21 chemotactic gradients [35,125–127]. Finally, a range of molecules associated with antigen presentation to T cells are upregulated, including the T cell adhesion molecule (CD54/ICAM-1), co-stimulatory molecules (CD80, CD83, CD86, and CD40) and T cell presentation complexes (MHC-I and II) [101,128–130]. As maturation drives the migration of DCs to CD4 T cells (HIV’s primary target), this presents an ideal opportunity for the virus to gain transport from the site of infection to its target cell. Therefore, HIV has evolved mechanisms to subtly manipulate these cells to avoid viral degradation but maintain transport to T cells.

In 2004, Wilflingseder and colleagues demonstrated that HIV induced maturation of *in vitro* derived MDDCs through activating MAPK phosphorylation, driving CCR7 upregulation and subsequent DC migration towards lymphatic-homing chemokines. The group also showed that HIV exposure upregulated maturation/co-stimulatory markers CD83 and CD86 and that these mature MDDCs transferred HIV to CD4 T cells [131]. In a series of studies from 2006 to 2016, the Cunningham/Harman group built upon these observations by showing that HIV manipulates typical DC/LC maturation in several critical ways and, importantly, demonstrated many of these processes using *bona fide* tissue-derived LCs. In response to HIV exposure, they showed that *in vitro* MDDCs and *bona fide* LCs downregulated CLRs, including MR and DC-SIGN, whilst upregulating CCR7, CD54, CD80, CD83, and CD86 [132]. Secondly, they demonstrated that HIV induced partial maturation via two independent mechanisms, one mediated by viral binding and the other by microvesicles released alongside HIV virions [133,134]. Thirdly, they showed that HIV interfered with *in vitro* MDDC lysosomal enzyme expression and function [67]. Finally, they showed that the virus significantly manipulates the interferon system by shutting down interferon expression by blocking TBK1 signalling while at the same time, switching on a small subset of interferon-stimulated genes (ISGs) via IRF1 which aid in viral replication [135–138].

These later findings highlight the complexity of IFN dynamics in DC-mediated HIV transmission—with observed differences between both human versus primate models as well as *in vivo* versus *in vitro* experimentation. For example, Nasr and colleagues reaffirmed earlier observations by Harman and colleagues, showing that *in vitro* MDM HIV challenge led to upregulation of ISGs, such as viperin, which has been shown to inhibit viral production in MDMs, potentially contributing to noncytopathic HIV-1 infection. Importantly, this occurred in two distinct phases of the viral life cycle, with both operating independently of true IFN signalling [137,138]. Similarly, Rodriguez-Garcia and colleagues demonstrated that DC2 isolated from the human female reproductive tract upregulated immune response genes, including ISGs, following HIV exposure, yet exhibited minimal or suppressed type I IFN expression [27,139]. DC2 also upregulated inflammasome-related genes. In contrast, *in vivo* MDDCs upregulated genes involved in secretory antimicrobial responses [27]. This data contrasts with studies reporting robust IFN-α responses to HIV infection in both humans and nonhuman primates, often with a delayed detectability in plasma [140–143]. However, these responses have largely been attributed to pDC activation, which is known to produce substantial IFN upon HIV/SIV challenge *in vivo* [21,144,145]. These findings suggest that HIV mediated IFN responses differ significantly between DC subsets. While pDCs are potent producers of type I IFNs, other subsets may upregulate ISGs through IFN-independent pathways. This underscores the need for further *in vivo* classification of these mechanisms across tissue contexts, including inflammation. We direct the reader to a recent review on the subject by Warner van Dijk and colleagues [21].

Collectively, these and other studies suggest that HIV can induce DC maturation to a degree sufficient to promote migration to lymph nodes and activation of T cells, while simultaneously interfering with lysosomal processing—presumably to evade destruction. This allows the virus to be retained during transport to CD4 T cells, either within the mucosa or in draining lymph nodes, facilitating enhanced viral transfer and activation of inflammatory pathways. Notably, inhibiting interferon production reduces the protective mechanisms of neighbouring cells against HIV infection, thereby promoting viral spread. Additionally, the induction of a small subset of ISGs facilitates viral replication within the APCs thereby enabling persistent noncytopathic infection and sustained viral production.

## Immune kinetics of HIV harbouring DCs

Interestingly, the timeline of both innate and adaptive immune kinetics in response to HIV remains incompletely defined. A recent review by Martin-Moreno and Muñoz-Fernández highlighted a disconnect in the literature regarding DC maturation and first-phase HIV transmission. They noted that DCs typically take 12–24 h to migrate to lymph nodes, roughly the time limit for first-phase transmission—implying much of the virus would be degraded en route [60]. However, a 2022 study by Baharlou and colleagues demonstrated that HIV containing DCs interact with T cells within the colorectal mucosa within 2 h of topical exposure. Moreover, these DCs also transported the virus to tertiary lymphoid follicles located in nearby tissues [146]. Therefore, DCs may not be required to migrate to lymph nodes in order to transmit HIV to CD4 T cells.

Despite this potential for early transmission to CD4 T cells, *in vivo* nonhuman primate SIV studies indicate that adaptive immune activation is significantly delayed in response to SIV, allowing local and distal viral replication to establish a foothold across multiple tissues. As recently reviewed by Kazer and colleagues [147], HIV/SIV replication begins within hours in mucosal tissues, with dissemination to distal sites within 1–3 days, particularly within gastrointestinal and lymphoid tissues [147–149]. This early spread coincides with antiviral host responses that dampen CD4 and CD8 T cell activation [147,150]. As a result, adaptive responses do not emerge until around day 10, coinciding with peak systemic viremia. This delay in activation is important as innate and adaptive immune kinetics have been linked to SIV disease outcome and progression [151,152].

## Anogenital antigen-presenting cell subsets and HIV transmission

Early studies of APC-mediated HIV transmission focussed primarily on *in* *vitro*-derived APCs (DCs, LCs, and macrophages) due to the difficulties of isolating APCs from tissue. However, we now know that these differ greatly from *bona fide* tissue-resident APCs. The few early studies using human tissue APCs focussed on LCs due to their assumed exclusive localisation within the mucosal epithelium, and thus they were likely the first cells to be encountered by HIV during transmission. However, we now know that the anogenital epithelial layer also contains DCs [49,117], and that genital trauma [153], inflammation [154], microbiome disruption [155,156], and co-infections like herpes simplex virus compromise the epithelial barrier [157,158], increase T cell infiltration, and heighten HIV transmission risk. Therefore, APCs in the underlying lamina propria are also likely key HIV target cells [159,160]. In this section, we aim to integrate the principal HIV transmission mechanisms discussed above with our current understanding of specific human tissue-resident APCs in order to determine their role in the sexual transmission of HIV.

### Langerhans cells

**Fig 2:**
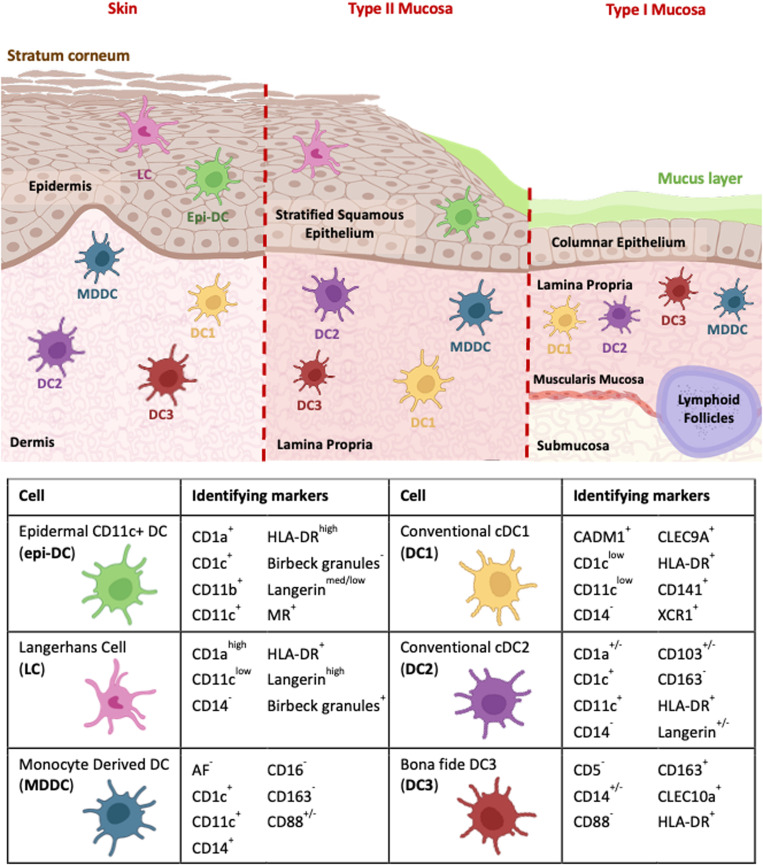
Human anogenital APC subsets and tissue compartment localisation. Skin and Type II mucosa of the distal anogenital tracts are characterised by a stratified squamous epidermis and epithelium, respectively. Both epidermis and stratified squamous epithelium contain LCs and Epi-DCs. Beneath these layers are the dermis (in skin) and lamina propria (in mucosa), which harbour DC1-3 and MDDCs under steady-state conditions. Type I Mucosa is located proximally along the anogenital tracts and is characterised by a superficial columnar epithelium overlying the deeper lamina propria, muscularis mucosa, and submucosa. Within Type I anogenital mucosa, DCs are primarily located in the lamina propria and include DC1-3 and MDDCs under steady-state conditions. The accompanying table summarises the anogenital DC surface markers that most reliably distinguish between anogenital tissues. Figures created with Biorender.com.

There is persistent controversy regarding LCs' importance in HIV transmission. Tissue LCs can be differentiated by their expression of CD45, CD1a, and langerin (Fig 2). Early studies suggested that LCs could transfer HIV to CD4 T cells [48,132,161], but in 2007, these conclusions were thrown into question when LCs were reported to be refractory to HIV infection by De Witte and colleagues. The group showed that LCs bind HIV and trafficked the virus to langerin-rich Birbeck granules, which degraded the virus [84]. However, subsequent studies using nontrypsin isolated human skin LCs demonstrated that they are capable of biphasic HIV transfer, with first phase mediated by langerin [83,85]. Additionally, langerin has since been shown to be expressed by anogenital DCs, especially those in the epithelium [26,49,71]. Therefore, early studies exploring the role of LCs in HIV transmission were inadvertently conducted on a mixture of DCs and LCs with the exception of electron microscopy studies, where LCs were defined via LC-specific Birbeck granules [162,163].

### Epithelial dendritic cells

Despite the early identification of Inflammatory Dendritic Epidermal Cells (IDEC) within inflamed skin in the 1990s [164], epidermal/epithelial DCs remained unstudied in the context of HIV transmission for almost two decades [164]. Then, in 2018, Pena-Cruz and colleagues discovered that HIV replicates and persists in Vaginal Dendritic Epithelial Cells (VDEC) [117]. Concurrently, Bertram and colleagues discovered CD11c^+^ epithelial DCs (epi-DCs) present in high proportions across every human anogenital tissue [49]. IDECs, VDECs, and epi-DCs have since been shown to be independent discoveries of the same cell, which we refer to as epi-DCs in this review [45]. Controversy remains as to whether epi-DCs differ to LC2s described by Liu and colleagues [44]. We refer the reader to a recent detailed review of the subject [47].

Like LCs, epi-DCs express the classic LC-defining markers HLA-DR, CD1a, and langerin. However, epi-DCs can be differentiated from LCs via: (i) CD206/MR expression; (ii) higher expression of CD1c, CD11b, CD11c; and (iii) lower expression of langerin and CD1a (Fig 2) [47,49,117]. Using RNA-seq, high-parameter flow cytometry, and microscopy, Bertram and colleagues demonstrated that epi-DCs were morphologically and transcriptionally indistinguishable from underlying dermal cDC2s, with only minor differences in surface protein expression observed. However, epi-DCs are functionally more efficient APCs than their dermal counterparts [49]. Of relevance to HIV transmission, epi-DCs are highly enriched in anogenital epithelium compared to LCs which are only minimally represented. Moreover, epi-DCs support productive infection of HIV [49,117] and are significantly more efficient at mediating both first- and second-phase transfer to CD4 T cells. Increased productive infection and second-phase transfer correlated with increased CCR5-mediated infection, however, it remains to be determined how increased first-phase transfer occurs as epi-DCs do not express the key HIV binding lectins that drive VCC formation, DC-SIGN and Siglec-1. It is tempting to speculate that this is mediated by langerin as a high proportion (up to 80%) of anogenital epi-DCs express this CLR, but at 10-fold lower levels than LCs. Therefore, further functional studies are required to elucidate the receptor that mediates HIV uptake by epi-DCs and onward transfer to T cells.

### Conventional dendritic cell 1 (DC1)

DC1s are the smallest population of DCs in steady-state human tissues [26,30] and are the only DCs that have proven capabilities to cross-present antigen to CD8 T cells [28–30,35]. Delineating DC1s from neighbouring sub-epithelial APCs has been challenging as markers that were used to discriminate them, such as CD141, are now known to be expressed by other DC subsets under certain conditions. However, DC1s can now be confidently discriminated across blood and tissue by their expression of XCR1, CADM1, and CLEC9A (Fig 2) [26,32,60,165–168]. DC1 is not thought to play a significant role in HIV transmission for two principal reasons. Firstly, they do not express HIV binding lectin receptors or entry co-receptors, CCR5, and CXCR4 [26], meaning that they cannot facilitate viral entry. Secondly, they express high levels of the innate retroviral restriction factor SAMHD1 [32]. Therefore, DC1s cannot mediate endocytic uptake of HIV and are resistant to HIV infection [169], rendering them incapable of mediating first- or second-phase transfer to CD4 T cells [26,71].

### Conventional dendritic cell 2 (DC2)

DC2s are the most abundant human tissue DC population and are present in all anogenital tissues [26,170,171]. They are best defined by their high expression of HLA-DR, CD11c, and CD1c (Fig 2). In skin, they also express CD1a, whereas in mucosal tissues they are known to express SIRPα [26,60,165,167,168]. While previously understudied, it is now clear that DC2 plays a significant role in HIV transmission. A proportion of them express langerin within tissue, with a notably higher proportion of langerin^+^ DC2s residing within anogenital mucosal lamina propria compared to skin dermis [26]. A 2021 study by Rhodes and colleagues showed that all anogenital DC2s can mediate both first- and second-phase transfer of HIV, but that the langerin^+^ population was more efficient at both transmission mechanisms. Potentially a result of their markedly increased expression of the HIV CLR, langerin and increased CCR5 expression [26]. A recent study by Parthasarathy and colleagues examined female genital tract APCs and HIV, in which they showed that DC2s expressed high amounts of the HIV entry receptors CD4, CCR5, and CXCR4 and that within 30 min of HIV exposure, they downregulated gene expression of the HIV restriction factor SAMHD1 and modulated pathways associated with activated inflammasomes and IFN responses [27].

### *In vivo* monocyte-derived dendritic cells (MDDC)

Reviewing the role of CD14-expressing DCs in HIV transmission requires a careful examination of the literature as CD14^+^ immune cell classification and nomenclature have undergone significant evolution within the last decade. Initial studies defined tissue-resident CD14+ cells as macrophages or DCs based on autofluorescence or nonautofluorescence, respectively [25]. In addition to the lack of autofluorescence, most early CD14 DC HIV transmission studies used DC-SIGN to define these cells, which is now known to be expressed by MDMs rather than MDDCs [26]. Most recently, an additional *bona fide* CD14^+^ DC population known as DC3 has emerged, adding further complexity to previous definitions of *in vivo* MDDCs. Current literature suggests that *in vivo* dermal/lamina propria MDDCs are best distinguished from MDMs, DC3s, and other sub-epithelial APCs by their positive surface expression of CD14, CD1c, CD11c, CD88, and notable lack of CD163 expression (Fig 2) [26,33,71,172]. Regarding the role of MDDCs in HIV transmission, in 2018, Trifonova and colleagues showed that *ex vivo* MDDCs were not only capable of HIV uptake and transfer to CD4 T cells but were far more efficient than MDMs at this process [51]. Perez-Zsolt echoed these findings in 2019 when they showed that *in vivo*-derived MDDCs from the cervical mucosa captured and transferred HIV-1 via Siglec-1 [38]. However, Rhodes and colleagues expanded upon these findings by showing that macrophages and MDMs express very high levels of Siglec-1, with MDDCs observed to express much lower amounts. They also demonstrated that both anogenital MDMs and MDDCs were equally capable of mediating first-phase transmission and that blocking transmission via Siglec-1 mainly blocked MDM uptake. Finally, MDDCs were more efficient at mediating second-phase infection, corresponding with higher CCR5 expression and therefore productive infection [26].

### Dendritic cell 3 (DC3)

The recent emergence of DC3s as an additional CD14+ DC has significantly altered the current DC landscape, not just for HIV research, but for immunology in general. However, not all DC3s are CD14+, with CD14+ DC3 believed to represent an inflammatory subset. For a comprehensive, contemporary review of the phenotypic presentation of DC3s within tissue, we refer readers to a recent review by Warner van Dijk and colleagues [21]. Currently, DC3s within tissue can be differentiated from other sub-epithelial APCs by their expression of CD163, and their lack of CD88 and CD5 (Fig 2) [33,172,173]. Given their recent discovery, the role of DC3s in facilitating HIV transmission is yet to be elucidated. The first publication objectively exploring a link between DC3s and HIV transmission was published in 2024 by Parthasarathy and colleagues who showed that in the female genital tract, DC3s express the genes encoding classical and nonclassical HIV co-receptors (CCR5, CD49d, and CX3CR1) [27].

## Concluding remarks

The advent of reliable antiretroviral therapies, combined with strong public health policies, has significantly reduced HIV’s impact in many higher-income countries. However, the pandemic continues to devastate sub-Saharan Africa and other lower-income regions, deepening global health inequalities. Despite decades of research, an estimated 1.3 million people acquired HIV in 2023, underscoring the urgent need for alternative, accessible prophylactic options [174].

Blocking initial infections requires a deeper understanding of early transmission events and the immune cells involved. While DCs have long been recognised as key players in HIV infection, advancements in high-parameter cell diagnostics have identified novel DC subsets with varying capacities to facilitate HIV transmission. Improved tissue isolation techniques have also revealed significant variability in DC subset proportions across human tissues, with anogenital tissues displaying a uniquely diverse DC landscape. These findings are crucial for ensuring physiological relevance in the pursuit of better prophylactic strategies.

Clarifying the role of DCs in HIV transmission and understanding how the virus exploits these cells has two major implications. Firstly, the design of better PrEP drugs, for example, those that block CLR-mediated HIV take by APCS or by disrupting HIV trafficking to CD4 T cells. Secondly, in vaccine design, as APCs are the first immune cells to encounter HIV and they then go on to drive adaptive immunity. For example next-generation mucosal vaccines could be tethered to specific lectin receptor ligands to targeting them to desired APC subsets to drive the best T cell-mediated immune responses.
